# ﻿Description of a new music frog (Anura, Ranidae, *Nidirana*) critically endangered in Taiwan

**DOI:** 10.3897/zookeys.1229.139344

**Published:** 2025-02-27

**Authors:** Chun-Fu Lin, Chunwen Chang, Masafumi Matsui, Chin-Chia Shen, Atsushi Tominaga, Si-Min Lin

**Affiliations:** 1 Taiwan Biodiversity Research Institute, Nantou, Taiwan; 2 Taiwan Forestry Research Institute, Taipei, Taiwan; 3 Graduate School of Human and Environmental Studies, Kyoto University, Kyoto, Japan; 4 School of Life Science, National Taiwan Normal University, Taipei, Taiwan; 5 Faculty of Education, University of the Ryukyus, Okinawa, Japan; 6 Graduate School of Engineering and Science, University of the Ryukyus, Okinawa, Japan

**Keywords:** Acoustic analysis, Iriomote Island, Ishigaki Island, morphology, *Nidiranaokinavana*

## Abstract

*Nidiranaokinavana* (Boettger, 1895) is a small-sized ranid species belonging to the East Asian genus *Nidirana* Dubois, 1992. Previous studies have indicated that this species was exclusively distributed on Ishigaki and Iriomote islands in the southern Ryukyus, as well as two extremely small wetland habitats in central Taiwan. Such a restricted distribution makes it one of the most endangered frog species in both Taiwan and Japan. By using molecular, morphological, and acoustic analyses, our study reveals significant divergence between the Taiwanese and Japanese clades, supporting the recognition of the Taiwanese clade as a distinct species, described herein as *Nidiranashyhhuangi***sp. nov**. Compared to *Nidiranaokinavana* sensu stricto from the southern Ryukyus, the *Nidiranashyhhuangi***sp. nov.** is characterized by a significantly smaller and non-overlapping body size, relatively longer forelimbs and hindlimbs, smaller internostril and interorbital distances, with a higher number of cross bands on thigh and shank. Acoustic analyses reveal that the *Nidiranashyhhuangi***sp. nov**. produces calls with a rapid tempo and higher pulse number, with a higher dominant frequency compared to the Japanese clade. Due to the extremely limited distribution of this species to two small sites on Taiwan, and continuing decline in quality of its habitat, we propose that it should be classified as Critically Endangered (CR) under the IUCN criteria. Immediate and comprehensive in situ and ex situ conservation actions are necessary to ensure the sustainable viability of the population.

## ﻿Introduction

*Nidirana* Dubois, 1992 is a small- to medium-sized ranid genus distributed in eastern and southeastern Asia. The majority of *Nidirana* species were discovered in the past decade, leading to a total of 19 species, including *N.okinavana* (Boettger, 1895); *N.pleuraden* (Boulenger, 1904); *N.adenopleura* (Boulenger, 1909); *N.daunchina* (Chang, 1933); *N.chapaensis* (Bourret, 1937); *N.lini* (Chou, 1999); *N.hainanensis* (Fei, Ye & Jiang, 2007); *N.nankunensis* Lyu, Zeng, Wang, Lin, Liu & Wang, 2017; *N.leishanensis* Li, Wei, Xu, Cui, Fei, Jiang, Liu & Wang, 2019; *N.yaoica* Lyu, Mo, Wan, Li, Pang & Wang, 2019; *N.guangdongensis* Lyu, Wan & Wang, 2020; *N.mangveni* Lyu, Qi & Wang, 2020; *N.xiangica* Lyu & Wang, 2020; *N.occidentalis* Lyu, Yang & Wang, 2020; *N.yeae* Wei, Li, Liu, Cheng, Xu & Wang, 2020; *N.guangxiensis* Mo, Lyu, Huang, Liao & Wang, 2021; *N.shiwandashanensis* Chen, Peng, Li & Liu, 2022; *N.noadihing* Boruah, Deepak & Das, 2023; and *N.chongqingensis* Ma & Wang, 2023.

**Figure 1.**
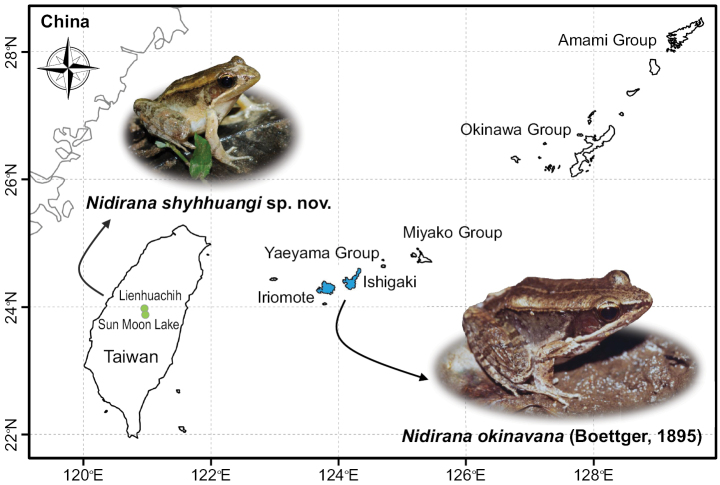
Distribution of *Nidiranaokinavana* sensu lato. *Nidiranaokinavana* (Boettger, 1895) is restricted to Ishigaki and Iriomote islands (photographed by the late Masataka Matsui), and *Nidiranashyhhuangi* sp. nov. is limited to two sites on Taiwan (photographed by CC). Although named after Okinawa, *N.okinavana* does not actually occur on that island.

In this paper, we describe a new species of *Nidirana* from Taiwan, which has long been regarded as conspecific with *N.okinavana*. *Nidiranaokinavana* was first described by Boettger in 1895 as “*Ranaokinavana*” (Boettger, 1895). The type locality was described as “Liukiu-Inseln, angeblich von Okinawa in der mittleren Gruppe”, referring to Okinawa Island in the central Ryukyus (Boettger 1895; Matsui 2007). However, because this species does not occur in the central Ryukyus, the species name was mistakenly applied to other frogs in Okinawa during the 20^th^ century. In 1985, Kuramoto described *Ranapsaltes* (Kuramoto, 1985) using specimens collected from Iriomote Island, southern Ryukyus. By comparing the type specimens, Matsui (2007) confirmed that *R.psaltes* is a synonym of *R.okinavana*, while the brown frogs in central Ryukyus were later given with two new species names (Matsui 2011). Lyu et al. (2017) further recognized the validity of *Nidirana* Dubois, 1992 as a distinct genus based on molecular, morphological, and bioacoustic evidence. Nowadays, the distribution of *N.okinavana* in the Ryukyus is confirmed to be only on Ishigaki and Iriomote islands of southern Ryukyus, which can also be collectively referred to as the Yaeyama Group (Fig. 1).

On the other hand, two *Nidirana* species are now recorded in Taiwan, one of which is *N.adenopleura*, the most widely distributed species among congeners in Taiwan and mainland China. In 1984, Shyh-Huang Chen discovered the second species during a collection trip to Lienhuachi (meaning “the Lily Pond”), Nantou County. This species represents morphology similar to *Ranapsaltes* in the southern Ryukyus, currently recognized as *N.okinavana*. Although aware of the potential differences between populations from Taiwan and the Ryukyus, and even designating the type series stored in the Department of Biology at National Taiwan Normal University (abbreviated as NTNUB in these specimens), Chen did not formally describe it as a new species. For the past forty years, this isolated population was regarded as the same species as *N.okinavana* (Chou 1994) and was confirmed to be distributed only in two sites in Nantou County: Lienhuachi and Xiangshan (an isolated valley near the famous landmark Sun Moon Lake) (Lin and Fu 2022). The extremely narrow distributional range with small population size makes it one of the most critically endangered amphibians in Taiwan (Lin et al. 2017; Lin and Fu 2022).

Due to the great geographic distance separating it from the nearest populations, the taxonomic status of *N.okinavana* in Taiwan has always been in doubt. Here we utilized mitochondrial DNA sequences and morphometric analyses to clarify that the population in Taiwan constituted a distinct species, *Nidiranashyhhuangi* sp. nov. We also discuss the potential ecological extinction risks faced by this species and the conservation status that should be assessed on a global scale.

## ﻿Materials and methods

### ﻿Genetic samples and DNA extraction

The sampling regimes comprise all the four currently known distribution sites: Ishigaki Island (*n* = 3) and Iriomote Island (*n* = 5) of southern Ryukyus, Japan; and two currently recognized sites from Taiwan (*n* = 31): Lienhuachi samples from a previous capture-mark-recapture program (*n* = 26); and Xiangshan (Sun Moon Lake) samples from tadpoles which faced a drying crisis in the wild (*n* = 5). All samples from Japan were collected before the relevant ordinance went into effect. Collection of samples or usage of preserved tissues from Taiwan were licensed by the Forestry and Nature Conservation Agency, Taiwan (No. 1080246108 and No. 1132400827).

Genomic DNA samples were extracted from preserved tissues using EasyPure Genomic DNA Spin Kit GT100 (Bioman, Taiwan) following the manufacture’s protocol. We suspended DNA in 60 μl EB buffer and stored vials at -20 °C refrigerator.

### ﻿DNA sequencing and phylogenetic analyses

We used polymerase chain reactions (PCRs) to amplify the following four mitochondrial fragments: 12S ribosomal RNA (12S), 16S ribosomal RNA (16S), cytochrome oxidase subunit I (COI), and cytochrome *b* (cyt*b*). Sequences, suggested annealing temperatures (T_m_), and references of the primers are listed in Suppl. material 1: table S1. The reactions were performed in a total of 20 μl volume containing 1 μl of DNA, 10 μl of 2× GoTaq® Green Master Mix (Promega, France), 0.2 μl of 10 mM forward and reverse primers, and 0.2–0.5 μl of 2.5 mM MgCl_2_. The PCR conditions consisted of denaturation at 94 °C for 4 min, followed by 40 cycles of denaturation at 94 °C for 30 sec, annealing at suggested T_m_ (Suppl. material 1: table S1) for 45 sec, and extension at 72 °C for 60 sec, with a final extension at 72 °C for 10 min. All PCR processes were performed by using a Biometra TOne Thermal Cycler (Analytic Jena, Germany). Electrophoresis was performed to assess the quality of PCR products on 1.2% agarose TBE gel, which was stained by FloroStain DNA Florescent Staining Dye (SMOBio, Taiwan).

PCR products were sequenced bidirectionally using an ABI 3730XL autosequencer (Genomics BioSci & Tech Corp., Taipei, Taiwan). Raw sequence data were assembled and edited using Sequencher 5.4.6 (Gene Codes Corporation, Boston, MA, USA) and aligned with the ClustalW (Thompson et al. 1994) implementation in MEGA 11 (Tamura et al. 2021).

### ﻿Phylogenetic analyses

Sequences of the four mitochondrial fragments of Taiwanese and Japanese samples were combined with all available *Nidirana* species in GenBank, as illustrated in Table 1. After alignment, the concatenated dataset was 2646 bp with 92 sequences. Population genetic diversity was estimated using DnaSP 6 (Rozas et al. 2017).

**Table 1.** GenBank Accession numbers, voucher numbers, and references of mitochondrial sequences used in this study.

Species	16S	COI	12S	cytochrome *b*	Sample No.	Reference
*N.shihuangi* sp. nov.	PQ367823	PQ358973	PQ367817	PQ390749	Lienhuachih06	This study
	PQ367822	PQ358972	PQ367816	PQ390748	Lienhuachih17	This study
	PQ367821	PQ358971	PQ367815	PQ390747	Lienhuachih21	This study
	PQ367820	PQ358970	PQ367814	PQ390746	Lienhuachih01–05 Lienhuachih07–16 Lienhuachih18–20 Lienhuachih22–29 Xiangshan01–05	This study
*N.okinavana*	NC022872	NC022872	NC022872	NC022872	N/A	Kakehashi et al. (2013)
	PQ367818	PQ358968	PQ367811		URE2381^1^, KUHE10076^2^	This study ^1^Iriomote; ^2^Ishigaki
	PQ367819	PQ358969	PQ367813		URE0086^2^, URE2044^2^, URE2128^1^	This study ^1^Iriomote; ^2^Ishigaki
	PQ367824	PQ358974	PQ367812		URE2124^1^, URE2365^1^, URE2380^1^	This study ^1^Iriomote; ^2^Ishigaki
*N.adenopleura*	MF807846	N/A			N/A	Lyu et al. (2017)
	MN946445	MN945201			SYS a007358	Lyu et al. (2020a)
	MN946446	MN945202			SYS a007359	Lyu et al. (2020a)
*N.chapaensis*	KR827710	N/A			N/A	Grosjean et al. (2015)
	KR827711	KR087625			MNHN 2000.4850	Grosjean et al. (2015)
*N.chongqingensis*	OQ846777	OQ843905			SWU0001408	Ma and Wang (2023)
	OQ846778	OQ843906			SWU0001435	Ma and Wang (2023)
	OQ846779	OQ843907			SWU0001439	Ma and Wang (2023)
*N.daunchina*	MF807822	MF807861			SYS a004594	Lyu et al. (2020a)
	MF807823	MF807862			SYS a004595	Lyu et al. (2020a)
*N.guangdongensis*	MN946406	MN945162			SYS a005767	Lyu et al. (2020a)
	MN946407	MN945163			SYS a005768	Lyu et al. (2020a)
*N.guangxiensis*	MZ677222	MZ678729			NHMG 202007001	Lyu et al. (2021)
	MZ677223	MZ678730			NHMG 202007002	Lyu et al. (2021)
	MZ677224	MZ678731			NHMG 202007003	Lyu et al. (2021)
	MZ677225	MZ678732			NHMG 202007004	Lyu et al. (2021)
	MZ677226	MZ678733			NHMG 202007005	Lyu et al. (2021)
*N.hainanensis*	MN946451	MN945207			SYS a007669	Lyu et al. (2020a)
	MN946452	MN945208			SYS a007670	Lyu et al. (2020a)
*N.leishanensis*	MN946453	MN945209			SYS a007908	Lyu et al. (2021)
	MN946454	MN945210			SYS a007195	Lyu et al. (2021)
	MZ677227	MZ678734			NHMG 202007021	Lyu et al. unpublished
	MZ677228	MZ678735			NHMG 202007022	Lyu et al. unpublished
	ON985176	ON968958			NNU 00694	Chen et al. (2022b)
	ON985177	ON968959			NNU 00769	Chen et al. (2022b)
	ON985178	ON968960			NNU 00770	Chen et al. (2022b)
	ON985179	ON968961			NNU 00810	Chen et al. (2022b)
	ON985180	ON968962			NNU 00917	Chen et al. (2022b)
*N.lini*	MF807818	MF807857			SYS a003967	Lyu et al. (2017)
	MF807819	MF807858			SYS a003968	Lyu et al. (2017)
*N.mangveni*	MN946424	MN945180			SYS a006310	Lyu et al. (2020a)
	MN946425	MN945181			SYS a006311	Lyu et al. (2020a)
	MN946432	MN945188			SYS a007990	Lyu et al. (2020a)
*N.nankunensis*	MF807838	MF807877			SYS a005717	Lyu et al. (2017)
	MF807839	MF807878			SYS a005718	Lyu et al. (2017)
	MF807840	MF807879			SYS a005719	Lyu et al. (2017)
*N.occidentalis*	MF807816	MF807855			SYS a003775	Lyu et al. (2020a)
	MF807817	MF807856			SYS a003776	Lyu et al. (2020a)
*N.pleuraden*	MT935671	MT932850			SYS a007717	Lyu et al. (2020b)
	MT935683	MT932858			SYS a007858	Lyu et al. (2020b)
*N.shiwandashanensis*	MZ787977	MZ782098			NNU00238	Chen et al. (2022a)
	MZ787978	MZ782099			NNU00239	Chen et al. (2022a)
*N.xiangica*	MN946433	MN945189			SYS a006491	Lyu et al. (2020a)
	MN946434	MN945190			SYS a006492	Lyu et al. (2020a)
	MN946435	MN945191			SYS a006493	Lyu et al. (2020a)
	MN946436	MN945192			SYS a007269	Lyu et al. (2020a)
*N.yaoica*	MK882276	MK895041			SYS a007020	Lyu et al. (2019)
	MK882277	MK895042			SYS a007021	Lyu et al. (2019)
	MK882278	MK895043			SYS a007022	Lyu et al. (2019)
*N.yeae*	MN295227	MN295233			CIBTZ20190608004	Wei et al. (2020)
	MN295228	MN295234			CIBTZ20190608005	Wei et al. (2020)
	MN295231	MN295237			CIBTZ20160714016	Wei et al. (2020)
*Babinaholsti*	NC022870	NC022870	NC022870	NC022870	N/A	Kakehashi et al. (2013)
*Babinasubaspera*	NC022871	NC022871	NC022871	NC022871	N/A	Kakehashi et al. (2013)

Phylogeny of *Nidirana* was constructed using maximum likelihood (ML) and Bayesian analysis. The best-fit nucleotide substitution model of the dataset was determined by ModelFinder Plus (Kalyaanamoorthy et al. 2017) according to the Bayesian information criterion (BIC), indicating TIM2e+R2 for 12s and 16s rRNA genes, and TN+F+G4 for COI and cyt*b*. ML tree was constructed using IQ-TREE 2.2.0 (Minh et al. 2020) with its branch supports assessed by 1,000 ultrafast bootstrap (UFBoot) replicates (Hoang et al. 2018), using the “-bnni” option to minimize the risk of overestimating branch supports due to severe model violations. Bayesian analysis was conducted using MrBayes 3.2.7 (Ronquist et al. 2012). Two independent runs of 5 × 10^7^ generations with eight MCMC chains each were conducted simultaneously, starting from random trees and resampling each tree every 1,000 generations. Converged Bayesian runs were combined after the exclusion of 25% burn-in, and a majority rule consensus tree was created with nodal confidence assessed by posterior probabilities. Finally, the values of statistical supports from ML bootstraps and Bayesian posterior probabilities were labelled on corresponding nodes.

### ﻿Adult measurements and morphology analysis

We took morphometric measurements from 19 Taiwanese and 18 Japanese specimens as listed in Table 2. Characters and measurements were modified from Lyu et al. (2017) and Wang et al. (2017), including: ; snout–vent length (**SVL**), the length between the tip of the snout to the cloaca ; snout–forelimb length (**SFL**), from tip of the snout to the anterior margin of the forelimb insertion on the body ; dorsal width (**DW**), the distance between the parallel dorsolateral folds ; forelimb length (**FLL**) , the sum of upper arm length (**UaL**) , lower arm length (**LaL**), and hand length measured from the wrist to the longest (3^rd^) finger (**HdL**) ; hindlimb length (**HLL**) , the sum of thigh length (**ThL**) , shank length (**ShL**) , and foot length from ankle to the longest (4^th^) toe (**FtL**) ; head length (**HL**) , the length between tip of snout to the posterior edge of tympanum; head width (**HW**) , the maximum width of the head on the level of mouth angles in ventral view; snout–nostril distance (**SND**) , from tip of snout to the nostril; snout length (**SNT**) , from tip of snout to the anterior corner of the eye; snout–tympanum distance (**STD**) , from tip of snout to the anterior corner of the tympanum; eye diameter (**ED**) , horizontal diameter of the eye; tympanum diameter (**TD**) , horizontal diameter of tympanum; internostril distance (**IND**) , minimum distance between nostrils; and interorbital distance (**IOD**) , minimum distance between upper eyelids. Bilateral characteristics were measured on both sides of the specimens, and the averages were used for further analysis. All measurements were taken by S.-M. L. with a digital caliper to the nearest 0.1 mm (Mitutoyo, Kanagawa, Japan).

**Table 2.** Specimens used for morphometric measurements.

Species	Specimen No.	Sex	Collection date (year/mouth/date)	Collector	Sample locality	Voucher deposition
*N.shyhhuangi* sp. nov.	NTNUB 149801^B^	F	1984/07/11	Shyh-Huang Chen	Lienhuachih	NTNU
	NTNUB 149802^B^	M	1984/07/11	Shyh-Huang Chen	Lienhuachih	NTNU
	NTNUB 149803^B^	M	1984/07/11	Shyh-Huang Chen	Lienhuachih	NTNU
	NTNUB 149804^B^	F	1984/07/11	Shyh-Huang Chen	Lienhuachih	NTNU
	**NTNUB 149805^A^**	M	1984/07/11	Shyh-Huang Chen	Lienhuachih	NTNU
	NTNUB 149806^B^	M	1984/07/11	Shyh-Huang Chen	Lienhuachih	NTNU
	NTNUB E861^B^	M	1986/10/11	Shyh-Huang Chen	Lienhuachih	NTNU
	NTNUB E862^B^	M	1986/10/11	Shyh-Huang Chen	Lienhuachih	NTNU
	NTNUB E863^B^	F	1986/10/11	Shyh-Huang Chen	Lienhuachih	NTNU
	NTNUB 166201	M	1989/08/28	Shyh-Huang Chen	Lienhuachih	NTNU
	NTNUB 166202	M	1989/08/28	Shyh-Huang Chen	Lienhuachih	NTNU
	NTNUB 166203	M	1989/08/28	Shyh-Huang Chen	Lienhuachih	NTNU
	NTNUB 166204	M	1989/08/28	Shyh-Huang Chen	Lienhuachih	NTNU
	NTNUB 166205	F	1989/08/28	Shyh-Huang Chen	Lienhuachih	NTNU
	NMNS 2377-3588	M	1993/08/19	Wen-Hao Chou	Lienhuachih	NMNS
	NMNS 2377-3589	M	1993/08/19	Wen-Hao Chou	Lienhuachih	NMNS
	NMNS 2377-3590	M	1993/08/19	Wen-Hao Chou	Lienhuachih	NMNS
	NMNS LW-W-01	M	N/A	Wen-Hao Chou	Lienhuachih	NMNS
	NMNS N/A	M	N/A	Wen-Hao Chou	Lienhuachih	NMNS
*N.okinavana*	NTNUB 149701	M	1980/10/07	Mitsuru Kuramoto	Iriomote	NTNU
	NTNUB 149702	M	1980/10/07	Mitsuru Kuramoto	Iriomote	NTNU
	NMNS 3430-04426	M	2000/05/23	Wen-Hao Chou	Ishigaki	NMNS
	NMNS 3438-04455	M	2000/05/24	Wen-Hao Chou	Iriomote	NMNS
	NMNS 3438-04456	M	2000/05/24	Wen-Hao Chou	Iriomote	NMNS
	NMNS 3466-14602	M	2000/05/26	Wen-Hao Chou	Ishigaki	NMNS
	NMNS 3466-14603	M	2000/05/26	Wen-Hao Chou	Ishigaki	NMNS
	URE 086	F	2010/9/3	Atsushi Tominaga	Ishigaki	URE
	URE 1307	M	2012/9/22	Atsushi Tominaga	Ishigaki	URE
	URE 1309	M	2012/9/22	Atsushi Tominaga	Ishigaki	URE
	URE 2044	M	2014/10/28	Atsushi Tominaga	Ishigaki	URE
	URE 2128	M	2015/3/16	Atsushi Tominaga	Iriomote	URE
	URE 2129	F	2015/3/16	Atsushi Tominaga	Iriomote	URE
	URE 2365	M	2015/910	Atsushi Tominaga	Iriomote	URE
	URE 2380	F	2015/9/11	Atsushi Tominaga	Iriomote	URE
	URE 2381	?	2015/9/11	Atsushi Tominaga	Iriomote	URE
	URE 9682	M	2024/7/27	Atsushi Tominaga	Iriomote	URE
	URE 9683	F	2024/7/27	Atsushi Tominaga	Iriomote	URE

A: holotype of *Nidiranashyhhuangi* sp. nov.; B: paratypes. NTNU: National Taiwan Normal University (School of Life Science), Taipei, Taiwan; NMNS: National Museum of Natural Science (Department of Life Science), Taichung, Taiwan; URE: Faculty of Education, University of the Ryukyus, Okinawa, Japan.

Principal component analysis (PCA) was performed using R package “ade4” (Dray and Dufour 2007) for morphometric dataset to examine the multivariate variation. A total of 20 characters were used in morphometric PCA. In addition to SVL, all the other measurements were either standardized by dividing by SVL (including standardized HL, SFL, DW, UaL, LaL, HdL, FLL, ThL, ShL, FtL, HLL); or by dividing by HL (including HW, SND, SNT, STD, ED, TD, IND, IOD).

### ﻿Acoustic data collection

Recordings of male breeding calls were conducted using a Sony PCM-D1 recorder and a Sony ECM-959A microphone at 48 kHz and 24-bit depth. To avoid the influence of temperature on call characteristics, we only analyzed calls recorded at the temperature between 25–27 °C. The analysis included calls from eight Taiwanese and seven Japanese individuals, with at least five calls per individual averaged for analyses. We compared their differences on call duration (sec), pulse number (n), pulse frequency (n/sec), and dominant frequency (Hz) using Wilcoxon rank-sum test.

### ﻿Tadpole measurements

Tadpoles were obtained from egg mass collected in the wild on 21 June 2013 and reared in the laboratory at 25–28 °C. Tadpole stages were determined according to Gosner (1960). Measurements were obtained by photographing tadpoles alongside a scale bar from the images at Gosner stage 30. The following ten morphometric characters were measured: total length (**TOL**), from the tip of the snout to the tip of the tail ; snout–vent length (**SVL**), from the tip of the snout to the posterior edge of the vent ; maximum body width (**BW**), from the dorsal side ; maximum body height (**BH**), from the lateral side ; tail length (**TAL**), from the base of vent to the tip of tail ; tail height (**TAH**), the maximum height between upper and lower edges of the tail ; tail base width (**TBW**), the maximum width of tail base ; snout length (**SNT**), from the tip of the snout to the anterior corner of the eye ; snout–spiracle length (**SS**), from the tip of the snout to spiracle ; interorbital distance (**IOD**), the minimum distance between the inner edges of the upper eyelids.

## ﻿Results

### ﻿Phylogenetic analysis and genetic diversity

**Figure 2.**
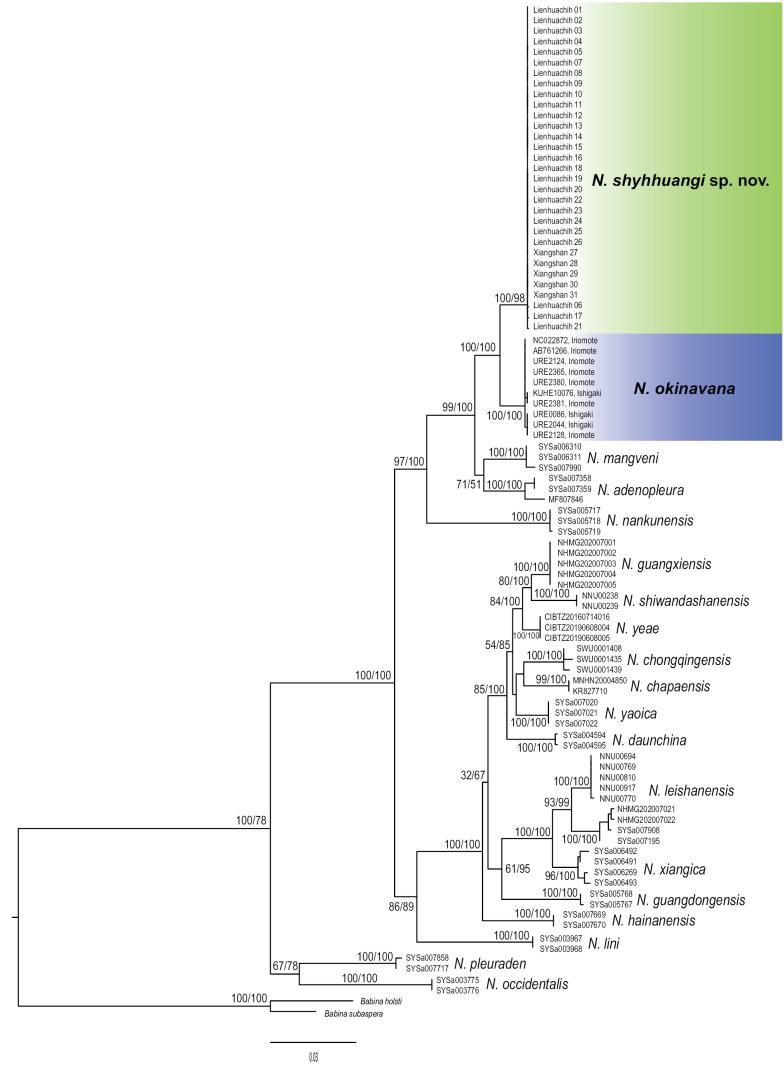
Maximum likelihood tree based on four mitochondrial fragments (2646 base pairs). Strong genetic divergence was detected between the clades from Taiwan (*Nidiranashyhhuangi* sp. nov.) and Yaeyama islands (*N.okinavana*). The values beside the nodes indicate statistical support from 1000 bootstrap replicates and Bayesian posterior probabilities.

Although there are some differences compared to the latest phylogeny using SNPs data (Lyu et al. 2024), the maximum likelihood tree incorporating the Taiwanese samples (Fig. 2) is similar to those from other studies based on mitochondrial data (Lyu et al. 2021; Chen et al. 2022a; Chen et al. 2022b). Basal lineages within the genus comprise *N.occidentalis* and *N.pleuraden*, while the remaining species form the other clades. *Nidiranaokinavana* sensu lato is closely related to *N.adenopleura*, *N.mangveni*, and *N.nankunensis*; and it is further separated as the Japanese clade (*N.okinavana* sensu stricto) and the Taiwanese clade (hereafter referred as *Nidiranashyhhuangi* sp. nov.) with both 100% bootstrap replicate and posterior probability. Genetic distances (*p*-distance; mean ± SD) between the two clades are 0.0210 ± 0.0045 in cyt*b*, and 0.0274 ± 0.0067 in COI.

In contrast to the considerable genetic divergence between *N.okinavana* sensu stricto and *Nidiranashyhhuangi* sp. nov., within-species polymorphism is low in both species. There is no genetic differentiation between frogs from Ishigaki and Iriomote islands. A total of two variable sites are detected, leading to three haplotypes from the ten available Japanese sequences. Similarly, there is no genetic differentiation between the two populations of *Nidiranashyhhuangi* sp. nov. in Taiwan, with three variable sites yielding four haplotypes. Haplotype diversity (Hd) and nucleotide diversity (π) are extremely low both in the Japanese clade (Hd = 0.689; π = 0.00055) and in the Taiwanese clade (Hd = 0.187, π = 0.00007).

### ﻿Morphological differences

**Figure 3.**
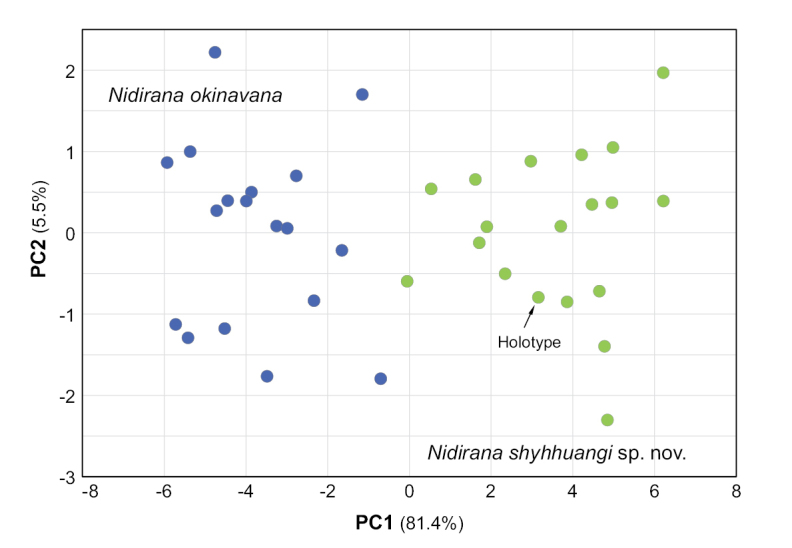
Principal component analysis (PCA) of *Nidiranashyhhuangi* sp. nov. and *N.okinavana*. PC1 and PC2 contributed to 81.4% and 5.5% variation, respectively.

**Figure 4.**
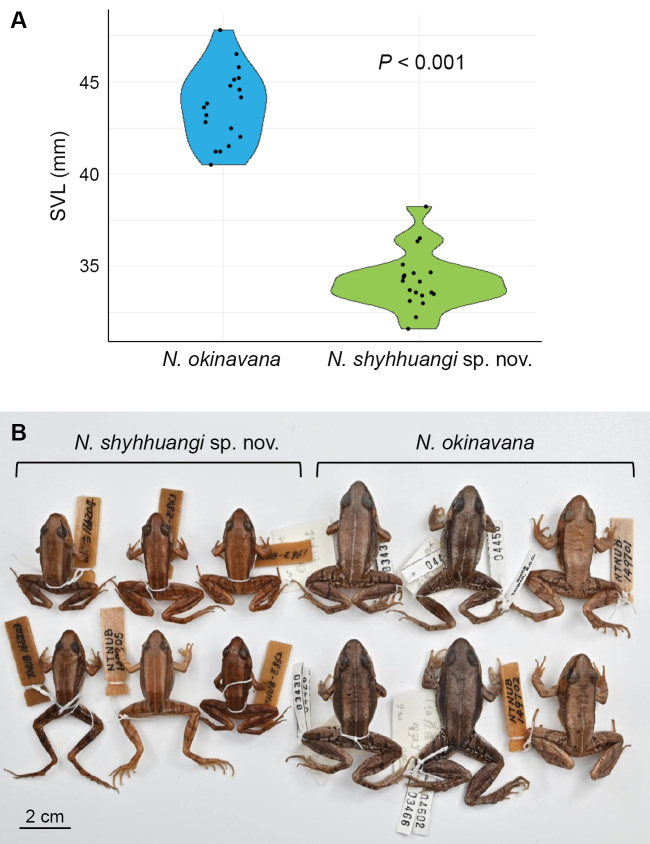
The body size of *Nidiranashyhhuangi* sp. nov. is significantly smaller than that of *N.okinavana* (Wilcoxon rank-sum test, *P* < 0.001). Photographed by Chih-Wei Chen.

PCA analysis using morphometric data indicated prominent morphological differentiation between *Nidiranashyhhuangi* sp. nov. and *N.okinavana* (Fig. 3). PC1 and PC2 contributed to 81.4% and 5.5% variation, respectively. Compared to *N.okinavana* (SVL = 43.7 ± 1.7 mm, mean ± SD; range 40.5–47.8 mm; *n* = 18), *Nidiranashyhhuangi* sp. nov. (SVL = 34.25 ± 1.54 mm; range 31.6–38.3 mm; *n* = 19) has a significantly smaller and non-overlapping body size (Wilcoxon rank-sum test, *P* < 0.001) (Fig. 4). When adjusted with body size, *Nidiranashyhhuangi* sp. nov. has a significantly longer snout–forelimb length (SFL/SVL; *P* < 0.01); a longer upper arm length (UaL/SVL; *P* < 0.001) and lower arm length (LaL/SVL; *P* < 0.001) which leads to a longer forelimb length (FLL/SVL; *P* < 0.001); a longer shank length (ShL/SVL; *P* < 0.01) and foot length (FtL/SVL; *P* < 0.05) which leads to a longer hindlimb length (HLL/SVL; *P* < 0.05). For the characters on head, *Nidiranashyhhuangi* sp. nov. has a shorter internostril distance (IND/HL; *P* < 0.001), a shorter interorbital distance (IOD/HL; *P* < 0.001), and a marginally larger relative tympanum diameter (TD/HL; *P* = 0.0569) (Suppl. material 1: table S2, fig. S1).

**Figure 5.**
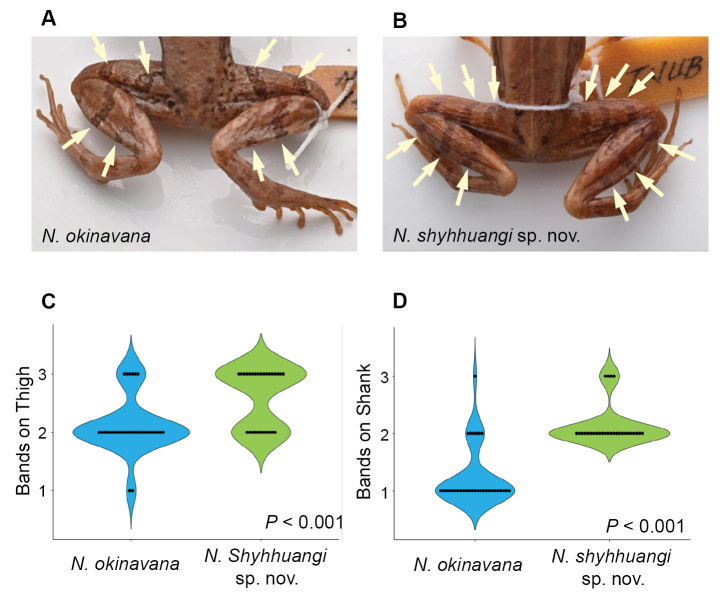
Comparison of transverse bands on the thigh and shank. Compared to **A***Nidiranaokinavana***B***Nidiranashyhhuangi* sp. nov. usually has one additional transverse band on both the thigh (**C** Wilcoxon rank-sum test, *P* < 0.001) and the shank (**D** Wilcoxon rank-sum test, *P* < 0.001). Photographed by Chih-Wei Chen.

In addition to morphometric data, we also observed differences in the number of transverse bands on the thigh and shank (Fig. 5). Most individuals from *N.okinavana* (75.0%) have two transverse bands on the thigh, while a smaller proportion (18.8%) has three and occasionally (6.2%) one (mean ± SD = 2.13 ± 0.49). In contrast, the majority of the *Nidiranashyhhuangi* sp. nov. (60.7%) has three bands, with a lower proportion (39.3%) having two (mean ± SD = 2.61 ± 0.50). On the shank, *N.okinavana* typically has only one complete transverse band (78.1%), while other markings do not form a complete band; only a lower proportion exhibits two (18.8%) or three (3.1%) bands. In contrast, *Nidiranashyhhuangi* sp. nov. consistently displays two (85.7%) or three (14.3%) bands. Overall, *Nidiranashyhhuangi* sp. nov. usually has one additional transverse band on both the thigh (Wilcoxon rank-sum test, *P* < 0.001; Fig. 5C) and the shank (Wilcoxon rank-sum test, *P* < 0.001; Fig. 5D) compared to *N.okinavana*.

### ﻿Acoustic differences

**Figure 6.**
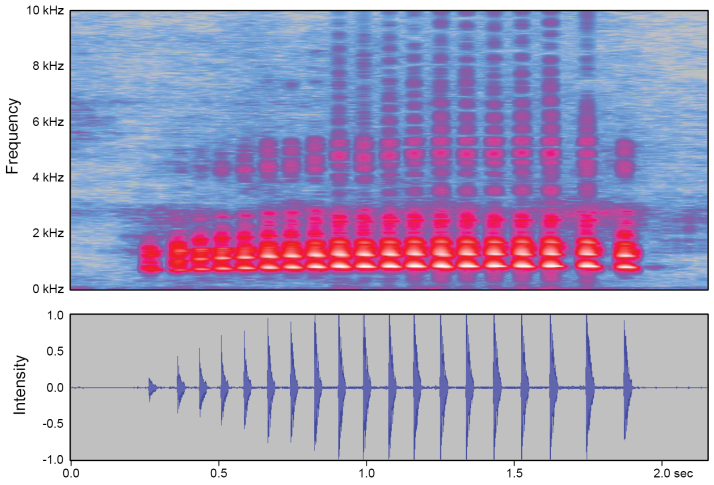
Mating calls of *Nidiranashyhhuangi* sp. nov. comprise quick, continuous, and regular pulses lasting 1.5–2.7 sec (1.808 ± 0.285 sec). Majority (> 85%) of a single call comprises 16–30 pulses (19.7 ± 5.0), with some cases reaching 32 pulses. Dominant frequency of the calls ranges between 800–900 Hz (840.5 ± 24.7).

Call characteristic from a total of eight *Nidiranashyhhuangi* sp. nov. and seven *N.okinavana* individuals were compared to evaluate their call differences (Table 3, Fig. 6). At the temperature between 25–27 °C, call durations are similar between *Nidiranashyhhuangi* sp. nov. (1.808 ± 0.285 sec; *n* = 8) and *N.okinavana* (1.868 ± 0.155 sec; *n* = 7). However, *Nidiranashyhhuangi* sp. nov. exhibits more pulse numbers (19.7 ± 5.0) than that of *N.okinavana* (15.8 ± 1.6) (marginal significance, *P* = 0.0561), which also leads to a higher pulse frequency of the former (10.8 ± 1.0 pulses/sec vs. 8.4 ± 0.3 pulses/sec; *P* < 0.01). Additionally, *Nidiranashyhhuangi* sp. nov. has a significantly higher dominant frequency (840.5 ± 24.7 Hz) compared to *N.okinavana* (723.4 ± 63.7 Hz) (*P* < 0.01). In conclusion, *Nidiranashyhhuangi* sp. nov. produces calls that are rapid and urgent, characterized by a faster tempo and higher sound frequencies.

**Table 3.** Acoustic comparison between *Nidiranashyhhuangi* sp. nov. (*n* = 8) and *N.okinavana* (*n* = 7) at temperature 25 °C–27 °C.

Species	No.	Call duration (s)	Pulse number (n)	Pulse rate (n/s)**	Dominant frequency (Hz)**
*N.shyhhuangi* sp. nov.	1	1.706	19.1	11.2	860.3
	2	1.761	19.2	10.9	822.7
	3	1.529	15.6	10.2	886.4
	4	2.353	28.9	12.3	819.3
	5	2.121	25.9	12.2	854.3
	6	1.709	17.3	10.1	839.5
	7	1.546	15.0	9.7	816.0
	8	1.736	17.0	9.8	826.0
	mean ± SD	1.808 ± 0.285	19.7 ± 5.0	10.8 ± 1.0	840.5 ± 24.7
*N.okinavana*	1	1.725	14.2	8.2	759.6
	2	1.815	15.0	8.3	666.2
	3	1.691	14.0	8.3	821.2
	4	2.109	17.8	8.4	722.4
	5	1.825	14.8	8.1	726.8
	6	1.868	17.0	9.1	624.4
	7	2.041	17.6	8.6	743.0
	mean ± SD	1.868 ± 0.155	15.8 ± 1.6	8.4 ± 0.3	723.4 ± 63.7
Wilcoxon rank-sum test	*Z*	-0.8680	1.9095	3.1825	2.9511
	*P*	0.3843	0.0561	0.0015	0.0032

**: *P* < 0.01

Based on molecular, morphological, and acoustic differences, we treat the two clade as distinct taxa and described *Nidiranashyhhuangi* sp. nov. as a new species.

### ﻿Taxonomic account

#### ﻿ Nidirana shyhhuangi sp. nov.

Taxon classificationAnimaliaAnuraRanidae

A9BA94EA-A1C6-561C-8EB9-7B117C486010

https://zoobank.org/A182AA8A-F9EA-4D9A-A176-A8E21251C84F

Figs 7 , 8 , 9

##### Chresonymy.

*Ranapsaltes* — Chou 1994; Lue et al. 1999: 80–81; Pan 2000: 114–115; Lue et al. 2002: 80–81; Yang 2002: 50–51; Chen 2003: 46–47; Chen 2006: 48–49. *Ranaokinavana* — Matsui 2007; Yang et al. 2008: 68–69; Shang et al. 2009: 96–97; Yang 2009: 300–303. Hylarana (Nidirana) psaltes — Fei et al. 2010: 310. *Nidiranapsaltes* — Fei et al. 2012: 349. *Nidiranaokinavana* — Yang and Lee 2019: 92–95.

##### Type material.

***Holotype*.** NTNUB 149805 (Fig. 8), preserved in National Taiwan Normal University, Taipei, Taiwan. Adult male in a good state of preservation, collected by Shyh-Huang Chen on 11 July 1984 from Lienhuachih (23.92082°N, 120.88585°E), Yuchi Township, Nantou County, Taiwan. The environment of the type locality (Fig. 10A), at an elevation of ~ 670 m a.s.l., is characterized by small freshwater wetland areas surrounded by patchy bamboo forests and subtropical Ficus-Machilus hardwood forests, featuring a humid microhabitat in the understory. ***Paratypes* (*n* = 8).** NTNUB 149801 ♀, 149802 ♂, 148803 ♂, 149804 ♀, 149806 ♂ collected on 11 July 1984; and NTNUB E861 ♂, E862 ♂, E863 ♀ collected on 11 October 1986. All the above-mentioned specimens, in a good state of preservation, were collected from Lienhuachih by Shyh-Huang Chen and preserved in National Taiwan Normal University, Taipei, Taiwan (Table 2).

##### Other material examined

**(*n* = 10).** NTNUB 166201 ♂, 166202 ♂, 166203 ♂, 166204 ♂, 166205 ♀ collected on 28 August 1989 by Shyh-Huang Chen; NMNS 2377-3588 ♂, 2377-3589 ♂, 2377-3590 ♂ collected on 19 August 1993 by Wen-Hao Chou; NMNS LW-W-01 ♂ and an unnumbered specimen (♂) collected by Wen-Hao Chou with the collection date unknown. All of the above specimens were collected from Lienhuachih (Table 2).

##### Etymology.

The specific epithet of the new species *shyhhuangi* is a Latinized patronymic noun in genitive case. It is dedicated to Prof. Shyh-Huang Chen, a herpetologist and arachnologist who first discovered this species in the early 1980s. We propose the common name “Yuchi music frog” in English to demonstrate the only two sampling sites of the species in Yuchi (meaning the “fish pond”) Township, or “魚池琴蛙” (pronounced as Yú-Chí-Qín-Wā) in Mandarin for this species.

##### Diagnosis.

*Nidiranashyhhuangi* sp. nov. is characterized by a combination of the following characters: (1) a small-sized ranid, body moderately slender; (2) SVL of preserved specimens in adult males 31.6–36.5 mm (mean ± SD = 33.8 ± 1.2 mm, *n* = 15), females 33.7–38.3 mm (35.9 ± 1.9 mm, *n* = 4); (3) head triangular, slightly longer than wide; (4) snout comparatively long, triangular in dorsal view with a slightly obtuse tip, moderately pointed in profile; (5) canthus rostralis distinct, contacting with the upper margin of nostril; (6) loreal region dark brown, extending posteriorly over eye and beyond tympanum; (7) upper lip pale white, white line extending posteriorly, forming a white stripe from below nostril to base of forelimb; (8) iris golden above canthus rostralis, dark brown below; (9) tympanum dark brown and conspicuous, dark zone extending posteriorly forming a trapezoid shape; (10) males with a single subgular vocal sac; (11) dorsum brown or yellowish-brown, sometimes gray; distinct vertebral stripe present; spinules on dorsal skin absent; (12) dorsolateral fold prominent, forming a distinct color boundary between dorsum and lateral body; (13) upper half of lateral body dark brown, lower half light brown; (14) jaw, throat, chest, and abdomen uniformly pale creamy yellow, generally lacking spots or patterns; (15) forelimb moderately long, pale brown; upper arm usually with one dark band at the base, forearm with one thin transverse band at the middle; (16) fingers slender, relative finger lengths II < IV < I < III; tips weakly dilated into discs, lateroventral groove absent on all fingers; free of webbing; (17) hindlimb relatively long, brown in color; thigh with two or three transverse dark bands, shank with two; (18) toes long and thin, relative toe lengths I < II < V < III < IV; tips weakly dilated into discs, lateroventral groove present on all toes; webbing partial, webbing formula I 1–1 II 1–1 III 2–2 IV 2–2 V.

##### Description of holotype.

Adult male in a good state of preservation (Fig. 8). Measurements of the holotype (left/right, all in mm): SVL 33.0; SFL 14.9/14.2; AGL 14.0/13.1; DW 9.1; UaL 8.9/8.5, LaL 7.2/7.8, HdL 7.9/8.1, leading to FLL 23.9/24.4; ThL 16.1/16.5, ShL 17.8/17.9, FtL 24.8/25.2, leading to HLL 58.7/59.5; HL 12.1/12.3; HW 11.2; SNT 6.2/6.1; ED 3.4/3.7; SND 2.9/2.9; STD 9.8/9.9; TD 2.9/2.8; IND 3.9; IOD 3.3.

Body moderately slender and elongated. Head triangular, as wide as body; slightly longer (HL/HW = 110.0%). Snout comparatively long (50.3% HL), triangular and slightly obtuse in dorsal view, moderately pointed in profile. Canthus rostralis distinct; loreal region flat; nostril round, lateral, upper margin in contact with canthus rostralis, closer to tip of snout (23.7% HL) than to eye (50.3% HL). Eye moderate sized, diameter 28.9% of HL, slightly larger than eye-nostril distance (26.3% HL); interorbital space flat, width 26.9% of HL and 29.5% of HW. Tympanum readily visible, rounded; diameter 23.3% HL and 80.7% of eye diameter; tympanic annulus conspicuous. Supratympanic fold absent.

Forelimb moderately long; UaL 26.3% SVL; LaL 22.7% SVL, HdL 24.1% SVL. Fingers slender, free of webbing, rounded in cross-section, no skin fringes on fingers. First finger well-developed, tip of all fingers slightly expended, width of finger tips ~ 110% of the thinnest diameter of phalanx, lateroventral groove absent on all fingers. Relative finger lengths: II < IV < I < III; length of finger II (3.42) 69.2% of finger III (4.94). Inner metatarsal tubercle prominent and outer metatarsal tubercle obscure; subarticular tubercles present, rounded. Hindlimb relatively long; FmL 49.4% SVL, TbL 54.0% SVL, FtL 75.6% SVL. Toes long, thin, tips of toes slightly flattened, width of toe tips ~ 120% of thinnest diameter of phalanx, lateroventral groove present on all toes. Relative toe lengths: I < II < V < III < IV; length of toe I (4.6) 29.0% of finger IV (16.0). Webbing partial, webbing formula I 1–1 II 1–1 III 2–2 IV 2–2 V. Inner metatarsal tubercle ovoid-shaped, present at base of first toe at preaxial side; outer metatarsal tubercle absent; subarticular tubercles prominent, rounded.

**Figure 7.**
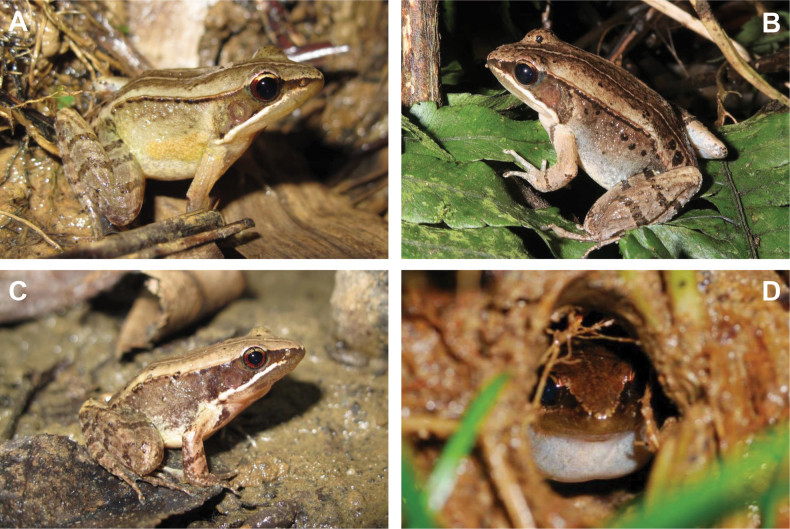
*Nidiranashyhhuangi* sp. nov. in life **A** an adult male with a pale yellowish-brown glandular ridge behind the base of the forelimb, which is diagnostic of the sexes **B** an adult female, with a more grayish coloration **C** a first-year juvenile, with a reddish lower iris color **D** a male calling in the nest showing its subgular vocal sac. Photographed by CFL (**A–C**) and CC (**D**).

***Color in life*.** Dorsum yellowish brown, darker near the midline and paler towards lateral edges; a thin but distinct pale vertebral stripe present (Fig. 7). Canthus rostralis and dorsolateral fold prominent, forming a distinct color boundary between dorsum and lateral body. Loreal region dark brown, extending posteriorly over eye and beyond tympanum. Upper lip pale white, white line extending posteriorly, forming a white stripe from below nostril to base of forelimb. Pupil deep black, iris golden above level of canthus rostralis, dark brown below, matching color of the loreal region. Tympanum dark brown, conspicuous, slightly translucent; dark zone extending posteriorly by approximately same width of tympanum diameter, forming a trapezoid shape. Dorsolateral fold darker than dorsum and flanks, displaying a fine but occasionally interrupted black line along fold. Upper half of lateral body similar to mid-dorsal coloration, dark brown; lower half paler, similar to lateral dorsum, pale brown. A slightly raised glandular ridge, pale yellowish brown in color, appearing as a mild swelling of skin, located behind base of forelimb. Jaw, chest, and abdomen uniformly pale creamy yellow, lacking spots or patterns. Forelimb pale brown; upper arm with one dark band at base, forearm with one thin transverse band at middle. Hindlimb brown, thigh with three transverse dark bands on both legs, shank with two; foot and toes with three bands on the left and four on the right.

**Figure 8.**
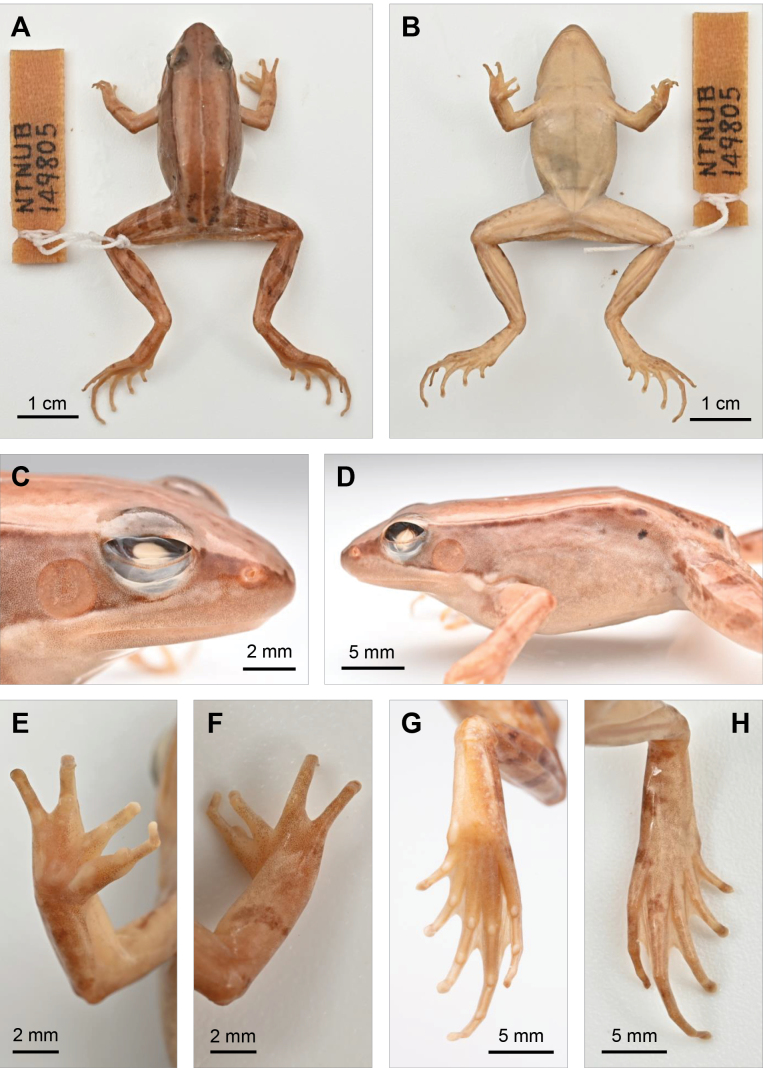
Holotype of *Nidiranashyhhuangi* sp. nov. NTNUB 149805, an adult male in a good state of preservation, collected by Shyh-Huang Chen on 11 July 1984 from Lienhuachih (23.92082°N, 120.88585°E), Yuchi Township, Nantou County, Taiwan. Photographed by Chih-Wei Chen.

***Color in preservative*.** Patterns in alcohol-preserved specimens show minimal change (Figs 4, 8). However, coloration fades slightly. Most brown tones become slightly paler, the yellow component of yellowish brown fades, and the white line on the upper lip becomes less distinct compared to live specimens.

***Variation*.** Some individuals are gray, a coloration that may be more common in females (Fig. 7B). The lower half of the iris in one-year juveniles is red or reddish brown (Fig. 7C). The number of transverse bands on the limbs varies: a majority of individuals (85.7%) have one transverse band on the forearm, while a minority (14.3%) has two. The number of bands on the thigh ranges from two (39.3%) to three (60.7%) (Fig. 5C), and on the shank, the bands are mostly two (85.7%), occasionally three (14.3%) (Fig. 5D). The number of bands on the foot is typically three (85.7%), occasionally four (14.3%).

***Sexual dimorphism*.** Sexual dimorphism within species of *Nidiranashyhhuangi* sp. nov. is recognized for glandular ridges and vocal sacs, both of which are present exclusively in males. Adult males can be distinguished from females by the presence of a pale yellowish-brown glandular ridge behind the base of the forelimbs, which is especially prominent during the breeding season (Fig. 7A). Compared to males, females tend to have a paler and more grayish coloration (Fig. 7B), while males typically have a darker body color. The males of *Nidiranashyhhuangi* sp. nov. have a single subgular vocal sac (Fig. 7D). A pair of vocal slits, located between the lower jaw musculature and epidermis, is present only in mature males and is absent in females. Additionally, males exhibit slightly raised nuptial pads, although they are not particularly prominent, and these pads are entirely absent in females.

Body size of both sexes overlap, but females are usually larger than males in both snout–vent length and body mass. SVL of preserved specimens in adult males 31.6–36.5 mm (mean ± SD = 33.8 ± 1.2 mm, *n* = 15), females 33.7–38.3 mm (35.9 ± 1.9 mm, *n* = 4). SVL of live individuals (C. Chang, unpublished data) in adult males 32.0–40.1 mm (35.5 ± 1.3 mm, *n* = 49), females 35.2–40.8 mm (38.3 ± 1.5 mm, *n* = 16). Body mass of live individuals (CC, unpublished data) in adult males 3.60–5.25 g (4.38 ± 0.42 g, *n* = 49), females 4.75–6.70 g (5.52 ± 0.51 g, *n* = 16).

***Comparisons*.***Nidiranashyhhuangi* sp. nov. could be distinguished from its most closely related congener, *N.okinavana*, by its significantly smaller and non-overlapping adult body size (Fig. 4). *Nidiranaokinavana* always exceeds 40 mm (*n* = 18 adults), whereas *Nidiranashyhhuangi* sp. nov. never exceeds 38.3 mm (*n* = 19 adults). *Nidiranaokinavana* usually has two transverse bands on the thigh and one on the shank; whereas *Nidiranashyhhuangi* sp. nov. typically has three bands on thigh and two on the shank (Fig. 5). *Nidiranashyhhuangi* sp. nov. could be further distinguished by a relatively longer snout–forelimb length (SFL/SVL), upper arm length (UaL/SVL), lower arm length (LaL/SVL), forelimb length (FLL/SVL), shank length (ShL/SVL), foot length (FtL/SVL), and hindlimb length (HLL/SVL) (Suppl. material 1: fig. S1). Additionally, *Nidiranashyhhuangi* sp. nov. has relatively larger tympanum (TD/HL), shorter internostril distance (IND/HL), and shorter interorbital distance (IOD/HL) for characters at head (Suppl. material 1: fig. S1).

The smaller body size of *Nidiranashyhhuangi* sp. nov. also leads to differences in behavioral traits compared to *N.okinavana*. The new species is in the nest building group of the genus; the mud nest opening (Figs 7D, 10B) measures approximately 2 cm in diameter in the new species, while that of *N.okinavana* can reach up to 5–6 cm. The mean clutch size of *Nidiranashyhhuangi* sp. nov. is 39 eggs (maximum 51), whereas *N.okinavana* can have up to 65 eggs (Kuramoto 1985) or even 80 eggs (Utsunomiya and Utsunomiya 1983; Maeda and Matsui 1990; Matsui and Maeda 2018).

*Nidiranashyhhuangi* sp. nov. is sympatrically distributed with the much more abundant congener, *N.adenopleura*. Body size of the latter is significantly larger than both *Nidiranashyhhuangi* sp. nov. and *N.okinavana*, with SVL ranging from 45–70 mm in the Taiwanese population (Lue et al. 1999; Lin and Fu 2022). *Nidiranaadenopleura* usually has one additional (3 or 4) transverse band on the thigh, with more and clearer warts and black spots on its lateral skin. Dorsal coloration of *Nidiranashyhhuangi* sp. nov. is paler, with dark brown longitudinal stripes on either side of the pale yellow mid-dorsal line. The males of *Nidiranashyhhuangi* sp. nov. have a single subgular vocal sac (Fig. 7D), whereas that of *N.adenopleura* has a bilobed subgular sac.

Body size of *Nidiranashyhhuangi* sp. nov. is smaller than all other congeners, which makes it the smallest *Nidirana* of all. SVL of *Nidiranashyhhuangi* sp. nov. does not exceed 36.5 mm in males and 38.3 mm in females. On the other hand, most of the other *Nidirana* spp. exceeds 40 mm in both sexes, including *N.daunchina* (40.6–53.0 mm, Lyu et al. 2017), *N.guangdongensis* (50.0–59.3 mm, Lyu et al. 2020b), *N.guangxiensis* (40.2–51.0 mm, Lyu et al. 2021), *N.leishanensis* (49.5–56.4 mm, Li et al. 2019), *N.lini* (44.1–68.6 mm, Chou 1999; Lyu et al. 2017), *N.mangveni* (53.6–65.1 mm, Lyu et al. 2020b), *N.occidentalis* (44.5–61.3 mm, Lyu et al. 2020a), *N.pleuraden* (46.2–61.7 mm, Lyu et al. 2017; Lyu et al. 2020a), *N.shiwandashanensis* (46.2–50.8 mm, Chen et al. 2022a), *N.xiangica* (53.5–62.6 mm, Lyu et al. 2020b), *N.yaoica* (42.1–45.6 mm, Lyu et al. 2019), and *N.yeae* (41.2–44.7 mm, Wei et al. 2020).

SVL of *Nidiranashyhhuangi* sp. nov. overlaps with *N.chapaensis* (35.5–51.8 mm, Chuaynkern et al. 2010), *N.hainanensis* (32.8–44.4 mm, Fei et al. 2007), and *N.nankunensis* (33.3–39.5 mm, Lyu et al. 2017). However, the lateroventral groove is present on the fingers of *N.hainanensis*, and also present on all fingers except finger I in *N.chapaensis* and *N.nankunensis*. In contrast, the lateroventral groove is absent on the fingers of *Nidiranashyhhuangi* sp. nov.

In addition to body size, *Nidiranashyhhuangi* sp. nov. could be distinguished from *N.occidentalis* and *N.pleuraden* for dilated finger tips and toe tips; from *N.mangveni* for relative length of fingers (II < I < IV < III); from *N.adenopleura*, *N.guangdongensis*, *N.lini*, *N.mangveni*, *N.occidentalis*, *N.pleuraden*, and *N.xiangica* for absence of Spinules on dorsal skin. Comparisons among these species are listed in Table 4, with updated information of *N.okinavana* revised from Matsui and Maeda (2018) and this study.

**Table 4.** Comparison among *Nidirana* species, with updated information from Matsui and Maeda (2018) and this study.

	Species	SVL of males (mm)	SVL of females (mm)	Fingers tips	Lateroventral groove on fingers	Relative length of fingers	Toes tips	Lateroventral groove on toes	Tibio-tarsal articulation	Subgular vocal sacs	Nuptial pad	Spinules on dorsal skin	Nest construction	Tadpole labial tooth row formula	Calling	References
1	*N.shyhhuangi* sp. nov.	31.6–36.5	33.7–38.3	Dilated	Usually absent	II < I < IV < III	Dilated	Present	Snout tip	Present	Poorly one on finger I	Absent	Present	I: 1+1/1+1:II	13–32 fast repeated notes	This study
2	*N.okinavana*	40.5–44.8	45.2–47.8	Dilated	Present except finger I	II < I < IV < III	Dilated	Present	Eye center near nostril	Present	Poorly one on finger I	Absent	Present	I: 1+1/1+1:II	14–18 fast repeated notes	Matsui and Maeda (2018); this study
3	*N.adenopleura*	43.1–57.6	47.6–60.7	Dilated	Present except finger I	II < I < IV < III	Dilated	Present	Snout tip or eye-snout	Present	One on finger I	Entire or posterior	Absent	I:1+1/1+1:II or I:0+0/1+1:I	2–5 regular notes	Lyu et al. (2017, 2020b);
4	*N.chapaensis*	35.5–42.5	41.0–51.8	Dilated	Present except finger I	II < I = IV < III	Dilated	Present	Nostril	Present	Two on finger I	Absent or few above vent	Present	I:1+2/1+1:II	3 notes	Chuaynkern et al. (2010)
5	*N.chongqingensis*	41.8-43.3	?	Not dilated	Present	II < I < IV < III	Slightly dilated	Present	Eye or nostril	Present	One on finger I	Absent	?	?	?	Ma and Wang. (2023)
6	*N.daunchina*	40.6–51.0	44.0–53.0	Dilated	Absent or rarely present	II < I < IV < III	Dilated	Present	Nostril	Present	One on finger I	Absent	Present	I:1+1/1+1:II or I:1+1/2+2:I	2–5 notes containing a specific first note	Liu (1950); Lyu et al. (2017)
7	*N.guangdongensis*	50.0–58.4	55.3–59.3	Dilated	Present except finger I	II < I < IV < III	Dilated	Present	Nostril	Present	One on finger I	Entire	Absent	?	2–4 regular notes	Lyu et al. (2020a)
8	*N.guangxiensis*	40.2–47.6	49.9–51.0	Dilated	Present on fingers III and IV	II < I < IV < III	Dilated	Present	Nostril	Present	One on finger I	Absent	Present	I: 1+1/1+1:II	6–11 rapidly repeated regular notes	Lyu et al. (2021)
9	*N.hainanensis*	32.8–44.4	?	Dilated	Present	II < I < IV < III	Dilated	Present	Nostril	Present	Absent	Absent	Present	?	2–4 fast repeated double notes	Fei et al. (2007, 2009)
10	*N.leishanensis*	49.5–56.4	43.7–55.3	Dilated	Present	II < IV < I < III	Dilated	Present	Eye-snout	Present	Two on finger I	Absent	Absent	I: 1+2/1+1:II	1 single note	Li et al. (2019)
11	*N.lini*	44.1–63.1	57.7–68.6	Dilated	Present except finger I	II < I < IV < III	Dilated	Present	Beyond snout	Present	One on finger I	Posterior	Absent	I: 1+1/1+1:II	5–7 notes containing a specific first note	Chou (1999); Lyu et al. (2017)
12	*N.mangveni*	53.6–59.7	59.7–65.1	Dilated	Present on fingers III and IV	I < II < IV < III	Dilated	Present	Anterior corner of eye	Present	One on finger I	Entire or posterior	Absent	?	2–7 regular notes	Lyu et al. (2020a)
13	*N.nankunensis*	33.3–37.1	37.8–39.5	Dilated	Present except finger I	II < I < IV < III	Dilated	Present	Nostril	Present	One on finger I	Absent or few above vent	Present	I: 1+1/1+1:II	13–15 notes containing a specific first note	Lyu et al. (2017)
14	*N.occidentalis*	44.5–53.0	55.6–61.3	Not dilated	Absent	II < I < IV < III	Not dilated	Absent	Eye	Present	One on finger I	Posterior	Absent	?	3–5 regular notes	Lyu et al. (2020b)
15	*N.pleuraden*	46.2–52.3	46.9–61.7	Not dilated	Absent	II < I < IV < III	Not dilated	Absent	Nostril	Present	One on finger I	Posterior	Absent	I: 1+1/1+1:II	1–4 regular notes	Lyu et al. (2017, 2020b)
16	*N.shiwandashanensis*	46.2–50.8	48.3	Dilated	Present	II < IV < I < III	Dilated	Present	Eye	Present	One on finger I	Absent	?	I: 1+1/1+1:II	6–8 double notes	Chen et al. (2022)
17	*N.xiangica*	56.3–62.3	53.5–62.6	Dilated	Present	II < I < IV < III	Dilated	Present	Eye-snout	Present	One on finger I	Entire	Absent	?	2–3 notes containing a specific first note	Lyu et al. (2020a)
18	*N.yaoica*	42.1–45.6	?	Dilated	Present	II < I < IV < III	Dilated	Present	Nostril	Present	One on finger I	Absent	? (Probably present)	?	1–3 fast repeated regular notes	Lyu et al. (2019)
19	*N.yeae*	41.2–43.5	44.7	Dilated	Absent	II < IV < I < III	Dilated	Present	Eye	Present	One on finger I	Absent	? (Probably present)	I: 1+1/1+1:II	2–6 notes containing a specific first note	Wei et al. (2020)

***Call properties*.** Mating calls of *Nidiranashyhhuangi* sp. nov. comprises quick, continuous, and regular pulses lasting 1.5–2.7 sec (1.808 ± 0.285 sec; *n* = 8) (Table 3, Fig. 6; Suppl. materials S2–S4). The majority (> 85%) of a single call comprises 16–30 pulses, with some cases reaching 32 pulses. The dominant frequency of the calls usually ranges between 800–900 Hz (840.5 ± 24.7). Compared to the closely related *N.okinavana*, *Nidiranashyhhuangi* sp. nov. is characterized for its higher pulse number (19.7 ± 5.0 vs. 15.8 ± 1.6, *P* = 0.0561), higher pulse frequency (10.8 ± 1.0 pulses/sec vs. 8.4 ± 0.3 pulses/sec, *P* < 0.01), and higher dominant frequency (840.5 ± 24.7 Hz vs. 723.4 ± 63.7 Hz, *P* < 0.01).

***Tadpole description*.** Measurements at Gosner stage 30 (*n* = 1, in mm): TOL 41.5, SVL 13.9, BW 8.9, BH 6.2, TAL 26.8, TAH 6.7, TBW 3.6, SNL 3.1, SS 8.2, IOD 3.5.

**Figure 9.**
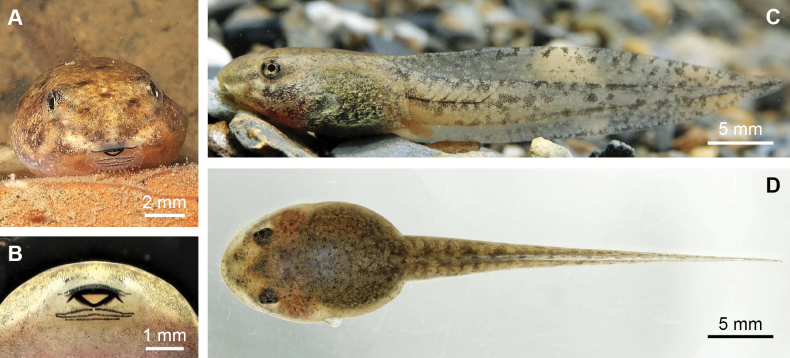
The tadpole of *Nidiranashyhhuangi* sp. nov. at Gosner stage 30. Photographed by CFL (**A, B, D**) and Da-Chaun Yeh (**C**).

In life, the body oval and dorsally flattened, with body width exceeding body height (BW/BH = 1.44) (Fig. 9). Both body and tail pale yellowish brown, covered with dense, minute golden dots, with several brown spots on dorsum and tail. Tail fusiform, ~ 1.93× snout–vent length, with a height comprising 25.0% of total tail length. Dorsal fin originates before base of tail. Eyes lateral, nostrils situated near snout. Spiracle located on left side of body, directed dorso-posteriorly. Labial tooth row formula 1:1+1/1+1:2, with lower lips bearing more labial papillae than upper (Fig. 9A, B).

After 40 days of growth, all tadpoles reached a maximum TOL of 53–54 mm (*n* = 30). By days 45–49, the tail had fully regressed, and the SVL of froglets measured 17.9–18.1 mm (*n* = 30). For comparison, *N.okinavana* tadpoles in Japan undergo metamorphosis at a SVL of ~ 20 mm, with a maximum TOL of ~ 70 mm (Maeda and Matsui 1990; Matsui and Maeda 2018), and froglets 20.0 ± 0.3 mm (mean ± SD) in SVL immediately after tail absorption (Kuramoto 1985).

##### Distribution and natural habitats.

*Nidiranashyhhuangi* sp. nov. is only known from two sites: Lienhuachih and Xiangshan (Sun Moon Lake). A past report from Jiaoxi Township in Yilan County (e.g., Shang et al. 2009; Fei et al. 2010; Fei et al. 2012; Yang and Li 2019) has been thoroughly investigated by local researchers, but this site could not be confirmed despite multiple surveys (S.-H. Chen, pers. comm., 2 July 2024 to SML).

**Figure 10.**
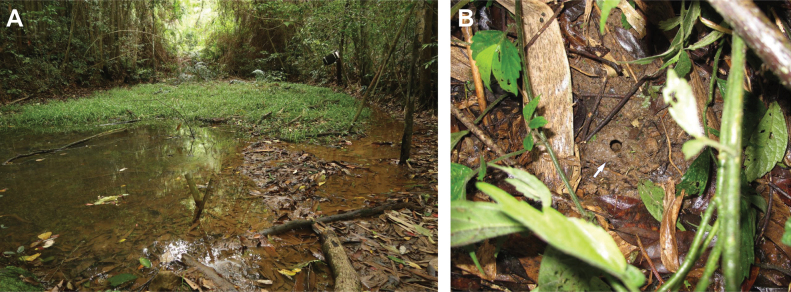
**A** type locality of *Nidiranashyhhuangi* sp. nov. in Lienhuachih (23.92082°N, 120.88585°E), Yuchi Township, Nantou County, Taiwan **B** a mud nest with an opening diameter of ~ 0.8 cm. The white arrow indicates the position of the opening. Photographed by CFL.

The habitat of Lienhuachih population (~ 670 m a.s.l.), discovered by Shyh-Huang Chen in 1984, is composed of ponds and patchy bamboo forests surrounded by typical low-elevation *Ficus*-*Machilus* forests (Fig. 10A). Water continuously seeps into the pond from the mountain hollow, keeping the pond at a stable water level. The substrate of the habitat remains moist and waterlogged throughout the year due to continuous water flow or hydrostatic pressure, with the soil being soft and highly absorbent. The soil texture in this area is yellow laterite soil with a topsoil of gray-brown sandy loam and subsoil composed of clay mixed with rock fragments (Koh et al. 1978; Lin and Fu 2022).

The second distribution site of *Nidiranashyhhuangi* sp. nov. was discovered from Xiangshan (~ 800 m a.s.l.) near the famous landmark Sun Moon Lake in 2005. The macrohabitat of this site comprises a valley terrain consisting of streams, dense forests, slopes, and forest paths (Lin and Fu 2022). The valley’s soil is interspersed with humus, making it very soft and moist. A subsurface water layer maintains the stream’s flow even during the dry season. Substrate in this area appears similar to reddish yellow earth indicative of laterization, likely consisting of gravelly clay, which is suitable for the nesting and reproduction of the frogs (Lin and Fu 2022).

Both habitats are situated in mountain hollows, which facilitate the collection of surrounding water sources. Coupled with the inflow of subsurface water, these habitats remain perennially waterlogged and moist even in the dry season, promoting the aggregation of the frogs.

##### Natural history notes.

*Nidiranashyhhuangi* sp. nov. is the only mud-nesting species among the ranid family in Taiwan (Figs 7D, 10B). Breeding season starts from April and lasts to late September. During the breeding season, male frogs select soil slopes within ~ 20 cm from the water to excavate mud nests, where they call from inside the nest to attract females. Newly built mud nests are shaped like pottery embryos, with an opening diameter of ~ 0.5–2 cm and an internal width of ~ 4 cm, often covered by fallen leaves or twigs. Unlike most frogs that lay eggs directly in water, the nest-building behavior reduces predation risk of the offspring, but requires significant energy expenditure. On average, it takes 2–3 hours for a male complete a nest (Lin and Fu 2022). The eggs are laid in cluster within the mud nest excavated by the male, with approximately 40 eggs per nest (range 31–51, mean ± SD = 39 ± 7, *n* = 6).

The eggs of the frog are encased in a transparent gelatinous substance and develop and hatch within the mud nests. The hatched tadpoles usually remain in the nest for a period of time, until heavy rain flushes the nests or water levels rise and allow the tadpoles to be released into adjacent streams or ponds. The tadpoles are brown in color, benthic, and have a larval period of 45–50 days at water temperatures of 26–29 °C (Lin and Fu 2022).

## ﻿Discussion

For frog species that call from mud nests, directly observing or documenting vocal sac morphology in the field is challenging. Relying solely on specimen examinations to determine the presence and type of vocal sacs can also lead to errors. Kuramoto (1985) initially described male *N.okinavana* as lacking vocal sacs. However, Matsui and Maeda (2018) re-examined specimens and revised this description, confirming the presence of a vocal sac in males. As the sister species of *N.okinavana*, *Nidiranashyhhuangi* sp. nov. also has a single vocal sac as illustrated in Fig. 7D. We also noted that further field observations would be essential to better understand some of the other secretive species.

Nuptial pads have been implicated in aiding the male to clasp the female during amplexus (Duellman and Trueb 1986). However, in species that mate within spatially confined mud nests, the necessity for firm clasping may be reduced. This might explain the relatively underdeveloped nuptial pads observed in male *Nidiranashyhhuangi* sp. nov. Given the challenges of observing reproductive behaviors in nest-building frog species, further research on the reproductive ecology of *Nidiranashyhhuangi* sp. nov. is needed.

Based on recent estimates (Lin and Fu 2022), the population size of mature *Nidiranashyhhuangi* sp. nov. in the Lienhuachih area during the breeding season is estimated to be between 60 and 90 individuals. The population size at Xiangshan is larger, with acoustic survey estimates indicating it is ~ 6× greater than that in Lienhuachih. Monthly acoustic monitoring since 2014 indicates that the Xiangshan population has remained relatively stable, while the Lienhuachih population shows a slight decline in annual trends (Lin and Fu 2022).

Before the Taiwanese clade was elevated as a valid species, this frog was classified as nationally critically endangered (CR) according to the “2017 Red List of Amphibians in Taiwan”, published jointly by the Endemic Species Research Institute and the Forestry Bureau (Lin et al. 2017). In Japan, *N.okinavana* is listed as vulnerable (VU) under Category II of the Japanese Ministry of the Environment’s threatened species list (Ministry of the Environment, Japan, 2020). International Union for Conservation of Nature (IUCN) classifies *N.okinavana* as an endangered species (EN) on a global scale (IUCN SSC Amphibian Specialist Group 2021). However, elevation of Taiwanese clade as a valid species makes it one of the rarest Arura around the world. Therefore, we recommend classifying *Nidiranashyhhuangi* sp. nov. as Critically Endangered (CR) which meets the IUCN criteria including B1a, B1b(iii), B1b(v),B2a, B2b(iii), and B2b(v) (IUCN Species Survival Commission 2000).

The major historical threats to *Nidiranashyhhuangi* sp. nov. in Taiwan include extremely limited habitat size, population isolation, and very small population size. The combined habitat area of the two existing populations in Lienhuachih and Xiangshan (Sun Moon Lake) totals ~ 0.015 km^2^, making them highly susceptible to extinction from climate events, habitat degradation, or other stochastic factors. The two distribution sites are isolated by ~ 8 km and separated due to the species’ specialized nesting behavior and preference for specific microhabitats, limiting their movements and interactions. The low population size also raises concerns about negative ecological effects, such as inbreeding depression (Lin and Fu 2022). Current and ongoing threats in these habitats include pollution from waste disposal, agricultural runoff, and human disturbances. Both populations face unregulated access to their habitats, with incidents of waste dumping, particularly around Sun Moon Lake, degrading the environment. Agricultural practices, such as deforestation, herbicide use, and pesticide runoff further deteriorate habitat quality. Human activity, such as trampling, can damage or compact soil, hindering the frog’s ability to dig nesting burrows, potentially crushing embryos or tadpoles (Lin and Fu 2022). Potential future threats include large- and small-scale developments that could alter water sources, extreme weather events from climate change, and competition from invasive species like the cane toad (*Rhinellamarina*), which has already been reported near the habitat of *Nidiranashyhhuangi* sp. nov. (Lin and Fu 2022) and has posed competitive risks to *N.okinavana* in Japan (IUCN SSC Amphibian Specialist Group 2021).

To protect this frog, monitoring land development around critical areas and population trends are essential. Water sources near Lienhuachih must be monitored to mitigate the effects of nearby lodging and camping developments that may alter groundwater flow. Expanding suitable habitats and establishing new ponds can help support population growth. Further efforts include establishing new habitats for ex-situ conservation to alleviate pressure on existing populations, conducting surveys of potential frog populations, and advancing ecological studies on reproductive behavior to support successful relocation and management strategies. The notorious *Polypedatesmegacephalus* and *Rhinellamarina*, both of which have become serious invasive species in Taiwan, have not yet invaded the habitat of *Nidiranashyhhuangi* sp. nov. However, if they successfully invade its habitat, they will become significant competitors or predators. We must closely monitor the areas surrounding the currently known distribution sites to prevent such an event from occurring. Finally, new projects have been proposed to use genome-wide markers, or even whole-genome sequencing, to compare the population genetics of *Nidiranashyhhuangi* sp. nov. and *N.okinavana*. This approach will help us understand their speciation history and assist in assessing the conservation genetics of these two species.

## Supplementary Material

XML Treatment for Nidirana shyhhuangi

## ﻿Additional information

### Author ORCIDs

Chun-Fu Lin https://orcid.org/0009-0003-6372-812X

Chunwen Chang https://orcid.org/0000-0002-1308-6945

Masafumi Matsui https://orcid.org/0000-0003-2032-2528

Chin-Chia Shen https://orcid.org/0009-0006-0203-9007

Atsushi Tominaga https://orcid.org/0000-0002-1495-7626

Si-Min Lin https://orcid.org/0000-0001-7080-706X

### Data availability

All of the data that support the findings of this study are available in the main text or Supplementary Information.

## Supplementary materials

Supplementary material 1 Additional information Data type docx Explanation note **table S1.** Primers, annealing temperatures (T_m_) in PCR, and references for the four mitochondrial fragments. **table S2.** Character differences (Wilcoxon rank-sum tests) between *Nidiranashyhhuangi* sp. nov. and *N.okinavana*. **fig. S1.** Morphological traits showing statistical significance between *Nidiranashyhhuangi* sp. nov. and *N.okinavana*. https://binary.pensoft.net/file/1264838This dataset is made available under the Open Database License (http://opendatacommons.org/licenses/odbl/1.0/). The Open Database License (ODbL) is a license agreement intended to allow users to freely share, modify, and use this Dataset while maintaining this same freedom for others, provided that the original source and author(s) are credited.Chun-Fu Lin, Chunwen Chang, Masafumi Matsui, Chin-Chia Shen, Atsushi Tominaga, Si-Min Lin

Supplementary material 2 Mating calls of *Nidiranashyhhuangi* sp. nov. (1) Data type wav https://binary.pensoft.net/file/1264839This dataset is made available under the Open Database License (http://opendatacommons.org/licenses/odbl/1.0/). The Open Database License (ODbL) is a license agreement intended to allow users to freely share, modify, and use this Dataset while maintaining this same freedom for others, provided that the original source and author(s) are credited.Chun-Fu Lin, Chunwen Chang, Masafumi Matsui, Chin-Chia Shen, Atsushi Tominaga, Si-Min Lin

Supplementary material 3 Mating calls of *Nidiranashyhhuangi* sp. nov. (2) Data type wav https://binary.pensoft.net/file/1264840This dataset is made available under the Open Database License (http://opendatacommons.org/licenses/odbl/1.0/). The Open Database License (ODbL) is a license agreement intended to allow users to freely share, modify, and use this Dataset while maintaining this same freedom for others, provided that the original source and author(s) are credited.Chun-Fu Lin, Chunwen Chang, Masafumi Matsui, Chin-Chia Shen, Atsushi Tominaga, Si-Min Lin

Supplementary material 4 Mating calls of *Nidiranashyhhuangi* sp. nov. (3) Data type wav https://binary.pensoft.net/file/1264841This dataset is made available under the Open Database License (http://opendatacommons.org/licenses/odbl/1.0/). The Open Database License (ODbL) is a license agreement intended to allow users to freely share, modify, and use this Dataset while maintaining this same freedom for others, provided that the original source and author(s) are credited.Chun-Fu Lin, Chunwen Chang, Masafumi Matsui, Chin-Chia Shen, Atsushi Tominaga, Si-Min Lin
