# Supplementary material for: ﻿Description of a new music frog (Anura, Ranidae, Nidirana) critically endangered in Taiwan
Source: Zookeys. 2025 Feb 27;1229:245–73. doi: 10.3897/zookeys.1229.139344 (PMC11886882; doi:10.3897/zookeys.1229.139344)
Supplement: Supplementary material 1 — Additional information [file zookeys-1229-245_article-139344__-s001.docx]

**Supplementary Material**

**Description of a new music frog (Anura, Ranidae, *Nidirana*) critically endangered in Taiwan**

Chun-Fu LIN [spring@tbri.gov.tw](mailto:spring@tbri.gov.tw)

Taiwan Biodiversity Research Institute, Nantou, Taiwan

Chunwen CHANG [chunwen@tfri.gov.tw](mailto:chunwen@tfri.gov.tw)

Taiwan Forestry Research Institute, Taipei, Taiwan

ORCID: <https://orcid.org/0000-0002-1308-6945>

Masafumi MATSUI [fumi@zoo.zool.kyoto-u.ac.jp](mailto:fumi@zoo.zool.kyoto-u.ac.jp)

Graduate School of Human and Environmental Studies, Kyoto University, Kyoto, Japan

ORCID: <https://orcid.org/0000-0003-2032-2528>

Chin-Chia SHEN [jinkergood@gmail.com](mailto:jinkergood@gmail.com)

School of Life Science, National Taiwan Normal University, Taipei, Taiwan

ORCID: <https://orcid.org/0009-0006-0203-9007>

Atsushi TOMINAGA* [tominaga@edu.u-ryukyu.ac.jp](mailto:tominaga@edu.u-ryukyu.ac.jp)

Faculty of Education, University of the Ryukyus, Okinawa, Japan

ORCID: <https://orcid.org/0000-0002-1495-7626>

Si-Min LIN* [lizard.dna@gmail.com](mailto:lizard.dna@gmail.com)

School of Life Science, National Taiwan Normal University, Taipei, Taiwan

ORCID: <https://orcid.org/0000-0001-7080-706X>

Chun-Fu LIN and Chunwen CHANG contributed equally to this work.

*: Corresponding authors: Atsushi TOMINAGA and Si-Min LIN

Running Title: A new *Nidirana* species

**Table S1.** Primers, annealing temperatures (T_m_) in PCR, and references for the four mitochondrial fragments.

| **Marker** | **Primer** | **Sequences (5’ – 3’)** | **T_m_** | **Reference** |
| --- | --- | --- | --- | --- |
| 12S rRNA | L1091 | CAAACTGGGATTAGATACCCCACTAT | 44 | Modified from Kocher et al. (1989) |
|  | H1478 | TGACTGCAAGGTGACGGGCGGTGTGT |  |  |
| 16S rRNA | L3975 | CGCCTGTTTACCAAAAACAT | 53 | Lyu et al. (2017) |
|  | H4551 | CCGGTCTGAACTCAGATCACGT |  |  |
| COI | Chmf4_modified | TTYTCTACTAATCAYAAAGACATTGG | 48 | Modified from Lyu et al. (2017) |
|  | Chmr4_modified | ACTTCWGGGTGYCCRAAGAATCA |  |  |
| Cytochrome *b* | Nidirana_cytbF1_1 | GATTAGAAGCYACTGCYAAAAGCC | 48 | This study |
|  | Cytb_R1_Rana | GGTTRGAWGAKCCMGTTTGGTGRAGGA |  |  |

**Table S2.** Character differences (Wilcoxon rank-sum tests) between *Nidirana shyhhuangi* sp. nov. and *N. okinavana*.

| Character | *N. okinavana* | *N. shyhhuangi* sp. nov. | *P*-value |
| --- | --- | --- | --- |
| SVL*** | 43.68 ± 1.72 | 34.24 ± 1.54 | *P* < 0.001 |
| SFL/SVL** | 0.3955 ± 0.0253 | 0.4262 ± 0.0269 | *P* < 0.01 |
| DW/SVL | 0.2623 ± 0.0131 | 0.2689 ± 0.0130 | *P* = 0.1419 |
| UaL/SVL*** | 0.2132 ± 0.0160 | 0.2482 ± 0.0135 | *P* < 0.001 |
| LaL/SVL*** | 0.2000± 0.0139 | 0.2238 ± 0.0129 | *P* < 0.001 |
| Hd/SVL | 0.2569 ± 0.0092 | 0.2508 ± 0.0144 | *P* = 0.1259 |
| FLL/SVL*** | 0.6700 ± 0.0278 | 0.7227 ± 0.0293 | *P* < 0.001 |
| ThL/SVL | 0.4696 ± 0.0223 | 0.4739 ± 0.0228 | *P* = 0.4797 |
| ShL/SVL** | 0.5040 ± 0.0146 | 0.5191 ± 0.0204 | *P* < 0.01 |
| FtL/SVL* | 0.7269 ± 0.0301 | 0.7502 ± 0.0252 | *P* < 0.05 |
| HLL/SVL* | 1.7002 ± 0.0566 | 1.7432 ± 0.0530 | *P* < 0.05 |
| HL/SVL | 0.3664 ± 0.0077 | 0.3689 ± 0.0118 | *P* = 0.1338 |
| HW/HL | 0.9528 ± 0.0472 | 0.9370 ± 0.0372 | *P* = 0.1594 |
| SND/SVL | 0.2376 ± 0.0131 | 0.2451 ± 0.0174 | *P* = 0.3275 |
| SNT/HL | 0.4485 ± 0.0158 | 0.4558 ± 0.0201 | *P* = 0.3583 |
| STD/HL | 0.7889 ± 0.0172 | 0.7909 ± 0.0312 | *P* = 0.6415 |
| ED/HL | 0.3058 ± 0.0226 | 0.3020 ± 0.0256 | *P* = 0.5990 |
| TD/HL | 0.2339 ± 0.0248 | 0.2462 ± 0.0172 | *P* = 0.0569 |
| IND/HL*** | 0.3391 ± 0.0170 | 0.2960 ± 0.0252 | *P* < 0.001 |
| IOD/HL*** | 0.2930 ± 0.0264 | 0.2435 ± 0.0178 | *P* < 0.001 |

*: *P* < 0.05; **: *P* < 0.01; ***: *P* < 0.001


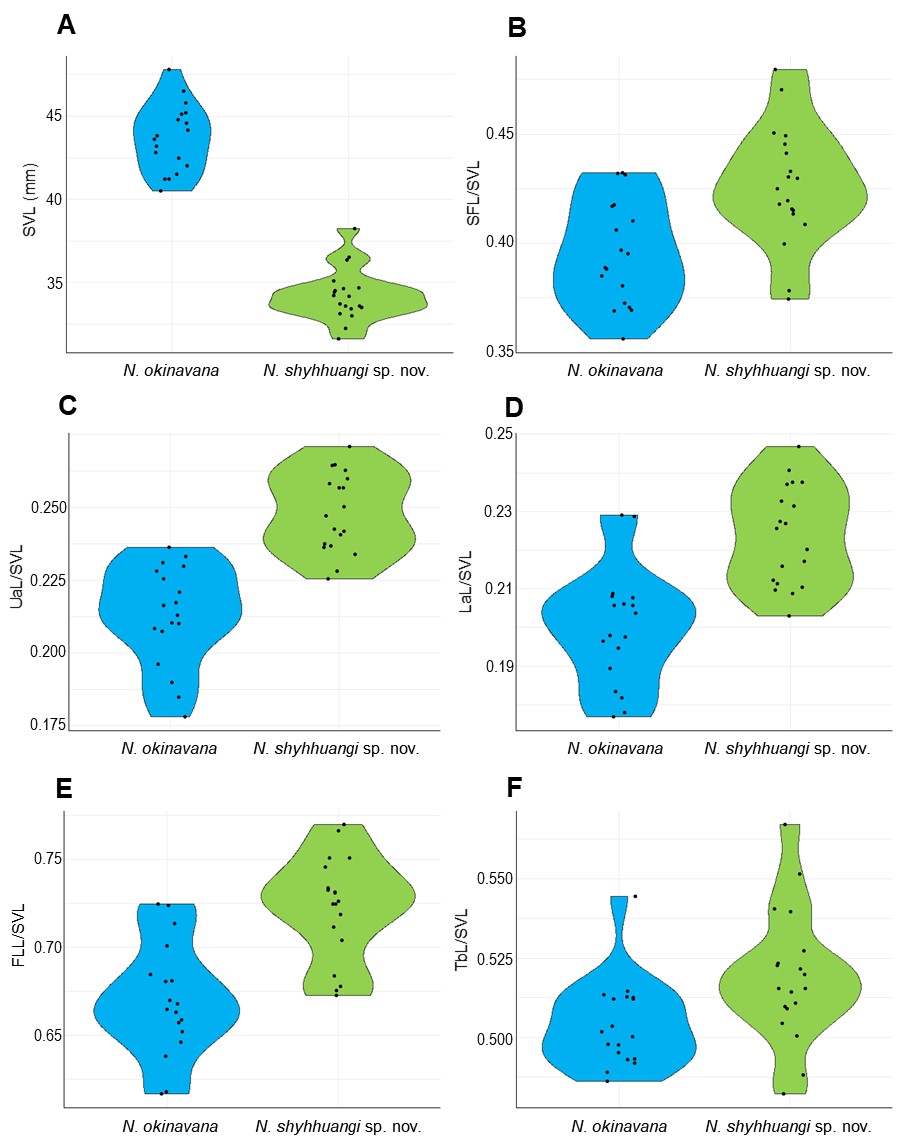


**Figure S1.** Morphological traits showing statistical significance between *Nidirana shyhhuangi* sp. nov. and *N. okinavana*, including (A) snout–vent length (SVL), *P* < 0.001; (B) relative snout–forlimb length (SFL/SVL), *P* < 0.01; (C) relative upper arm length (UaL/SVL), *P* < 0.001; (D) relative lower arm length (LaL/SVL), *P* < 0.001; (E) relative forelimb length (FLL/SVL), *P* < 0.001; and (F) relative shank length (ShL/SVL), *P* < 0.01.

**
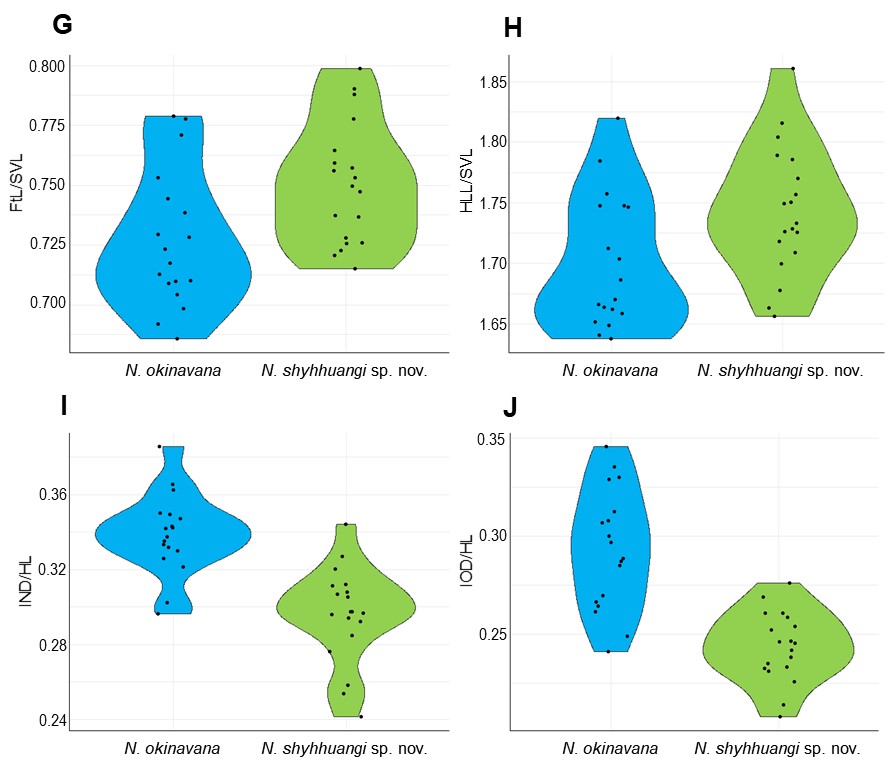
**

**Figure S1 (continued).** Morphological traits showing statistical significance between *Nidirana shyhhuangi* sp. nov. and *N. okinavana*, including (G) relative foot length (FtL/SVL), *P* < 0.05; (H) relative hindlimb length (HLL/SVL), *P* < 0.05; (I) relative internostril distance (IND/HL), *P* < 0.001; and (J) relative interorbital distance (IOD/HL), *P* < 0.001.
